# Low Cycle Fatigue Characteristics of Oxygen-Free Copper for Electric Power Equipment

**DOI:** 10.3390/ma14154237

**Published:** 2021-07-29

**Authors:** Takuma Tanaka, Togo Sugioka, Tatsuya Kobayashi, Ikuo Shohji, Yuya Shimada, Hiromitsu Watanabe, Yuichiro Kamakoshi

**Affiliations:** 1 Graduate School of Science and Technology, Gunma University, Kiryu 3768515, Japan; t191b046@gunma-u.ac.jp (T.T.); t211b041@gunma-u.ac.jp (T.S.); kobayashi.t@gunma-u.ac.jp (T.K.); 2 Analytic Simulation & Material Evaluation Center, Meidensha, Corp., Shinagawa 1418565, Japan; shimada-yu@mb.meidensha.co.jp (Y.S.); watanabe-hir@mb.meidensha.co.jp (H.W.); 3 Gunma Prefectural Industrial Technology Center, Maebashi 3792147, Japan; kamakoshi-yu@pref.gunma.lg.jp

**Keywords:** oxygen-free copper, tensile properties, low cycle fatigue properties, EBSD analysis, fatigue damage behavior

## Abstract

The effect of heat treatment on tensile and low cycle fatigue properties of the oxygen-free copper for electric power equipment was investigated. The heat treatment at 850 °C for 20 min, which corresponds to the vacuum brazing process, caused the grain growth and relaxation of strain by recrystallization, and thus, the residual stress in the oxygen-free copper was reduced. The tensile strength and 0.2% proof stress were decreased, and elongation was increased by the heat treatment accompanying recrystallization. The plastic strain in the heat-treated specimen was increased compared with that in the untreated specimen under the same stress amplitude condition, and thus, the low cycle fatigue life of the oxygen-free copper was degraded by the heat treatment. Striation was observed in the crack initiation area of the fractured surface in the case of the stress amplitude less than 100 MPa regardless of the presence of the heat treatment. With an increase in the stress amplitude, the river pattern and the quasicleavage fracture were mainly observed in the fracture surfaces of the untreated specimens, and they were observed with striations in the fracture surfaces of the heat-treated ones. The result of the electron backscattered diffraction (EBSD) analysis showed that the grain reference orientation deviation (GROD) map was confirmed to be effective to investigate the fatigue damage degree in the grain by low cycle fatigue. In addition, the EBSD analysis revealed that the grains were deformed, and the GROD value reached approximately 28° in the fractured areas of heat-treated specimens after the low cycle fatigue test.

## 1. Introduction

Recently, the demand of capacity enlargement of power supply used for power generation and electric transportation facilities has been increasing due to the increase of the electricity demand, steady supply of the electric power system, and low environmental impact. Conventionally, in order to consider the safety design of the electric power equipment, the stress amplitude in which the material can endure cyclic loading of approximately 10^7^ has been used as the practical fatigue limit [1]. In recent years, high output and high voltage have been required to the electric power equipment, which include a generator, a converter, an electric motor, and a circuit breaker. When high current flows in a power transmission system, the calorific value increases in contact materials. Thus, the mechanical stress loaded in the contact materials is increased due to the thermal expansion by heating. In such materials, it is also important to investigate the fatigue properties in a low cycle fatigue area by high stress.

Copper has been used for conductor parts of the electric power equipment due to its high conductivity [2,3]. Among them, the oxygen-free copper has many advantages such as gas tolerance at an electric discharge, low discharge of hydrogen gas, and vacuum deterioration tolerance [4], and thus, it has been used for the various types of electric power equipment. Generally, high cycle fatigue tests have been conducted in metals. Although similar tests have been also conducted in pure copper and copper alloys [5,6,7,8,9,10,11,12], there are a few experimental results of the oxygen-free copper in the low cycle fatigue region [5,10,11]. In addition, the oxygen-free copper parts used in the electric power equipment undergo a heat treatment process such as brazing [13], and thus, the investigation of fatigue properties with heat-treated specimens is required. In the brazing of copper and copper alloys, Ag-Cu-based filler metals [13,14] and Cu-P-based filler metals [15] have been generally used. Brazing with the Ag-Cu based filler metal is usually conducted on a vacuum condition [13,14], and the brazing cycle in the real product is relatively long time [16].

Thus, the aim of this study is to investigate the effect of heat cycle in the brazing process on the low cycle fatigue properties of the oxygen-free copper. Furthermore, the electron backscattered diffraction (EBSD) analysis was conducted to investigate the fatigue damage behavior of the heat-treated oxygen-free copper.

## 2. Materials and Methods

**Figure 1:**
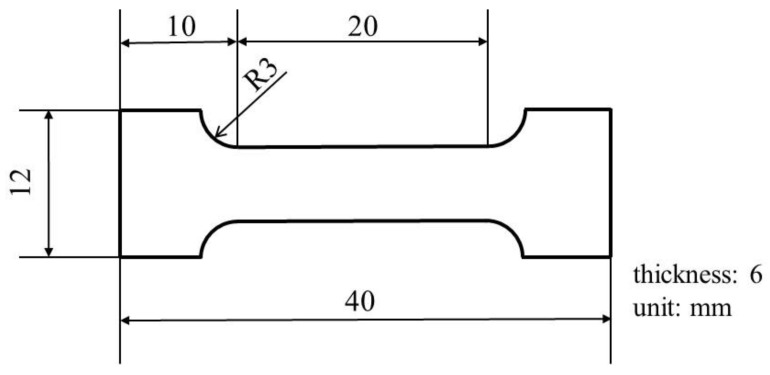
Shape and dimensions of C1020 specimen.

Table 1 shows the chemical composition of the C1020 oxygen-free copper used in this study. Figure 1 shows the shape and dimensions of the C1020 specimen. In this study, heat treatment that assumed the brazing process, which is applied to the vacuum brazing with Ag-Cu based filler metals, was conducted. The heat treatment was conducted at 850 °C for 20 min at a vacuum degree of 0.13 Pa. The heating period from room temperature (R.T.) to 850 °C was 7.2 h. The cooling from 850 °C to R.T. was conducted by natural heat radiation. The surface of the specimen was polished by a #800 waterproof abrasive paper. 

Residual stress measurement to the center of the specimen was conducted using a micro-area X-ray residual stress measurement system (AutoMATEII, Rigaku Corp., Tokyo, Japan). In the measurement, Cr Kα X-ray and a φ1 mm collimator were used. The residual stress at the loading direction, which is a longitudinal direction of the specimen shown in Figure 1, was measured.

A tensile test was conducted using a universal testing machine (5567, Instron Japan Co., Ltd., Kanagawa, Japan). In the test, a crosshead speed and test temperature were 4 mm/min and R.T., respectively. A fatigue test was conducted by load control using a triangular wave with a fatigue testing machine (EHF-E, Shimadzu Corp., Kyoto, Japan). The amplitude of load was 1.8–7.2 kN, which corresponds to the 50–200 MPa test stress estimated by the initial cross-section of the specimen. The frequency, stress ratio, and test temperature were 10 Hz, –1, and R.T., respectively. Fracture surfaces after the fatigue test were observed using a field emission scanning electron microscope (FE-SEM) (S-4300SE, Hitachi High-Tech Science, Inc., Tokyo, Japan).

To investigate the microstructures of the specimens before and after the fatigue test, the specimens were polished by #800–#4000 waterproof abrasive papers and were subsequently polished using 1 μm alumina powder suspension. Furthermore, sputtering etching by Ar milling was conducted to the polished surfaces using a cross-section milling machine (IB-19530CP, JEOL Ltd., Tokyo, Japan). An electron backscattering diffraction (EBSD) analysis was conducted to the surface of the specimen after Ar milling to investigate the fatigue damage behavior using an EBSD system (TSL MSC-2200, TexSEM Laboratories, Inc., Provo, UT, USA) equipped with the FE-SEM. In the EBSD analysis, the normal direction and the rolling direction were set to the normal direction to the surface of the specimen and the longitudinal direction of the specimen, respectively. For the initial microstructure, grain size measurement was also conducted. The specimen after polishing with the alumina powder suspension was etched for 120 s with an etching solution shown in Table 2. Thereafter, optical microscope observation was conducted using a laser microscope (VK-X150, KEYENCE Corp., Osaka, Japan). Using the obtained optical microscope images, the grain size was estimated by the intercept method [17] using image processing software (Image J, Wayne Rasband (NIH), Bethesda, MD, USA).

## 3. Results and Discussion

### 3.1. Initial Microstructure of Specimen

**Figure 2:**
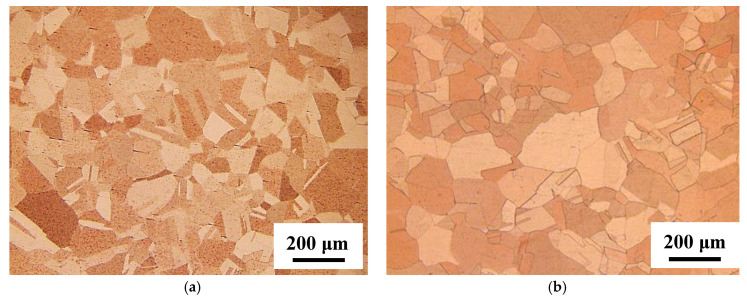
Optical microscope images after etching treatment. (**a**) Untreated; (**b**) heat-treated at 850 °C for 20 min.

**Figure 3:**
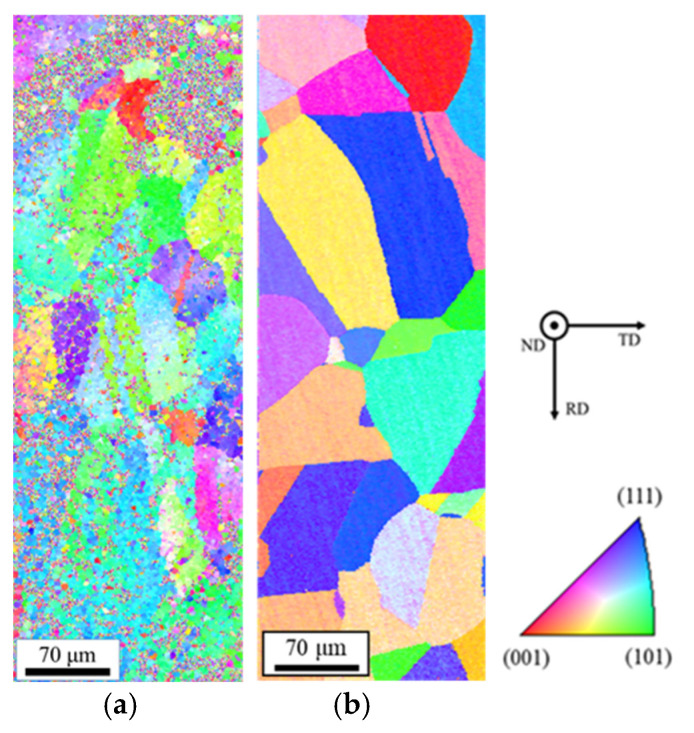
Inverse pole figure (IPF) maps estimated by electron backscattering diffraction (EBSD) analysis. (**a**) Untreated; (**b**) heat-treated at 850 °C for 20 min.

Figure 2 shows optical microscope images of the untreated specimen and the specimen with heat treatment at 850 °C for 20 min. Table 3 shows the average grain sizes estimated by the intercept method using Figure 2. It was confirmed that the grain growth occurs during heat treatment. Table 4 shows the measurement result of residual stress in the untreated specimen and the heat-treated one. In the untreated specimen, the compressive stress of 86.9 MPa to the longitudinal direction of the specimen remained. Table 4 indicates that the compressive stress was reduced from 86.9 to 70.3 MPa by heat treatment. It means that the grain growth by the heat treatment causes the relaxation of strain, and thus, the residual stress is reduced. Since the heat treatment temperature in this study was sufficient to cause a relaxation and secondary recrystallization, the grain growth was confirmed to be caused by the recrystallization. Figure 3 shows inverse pole figure (IPF) images estimated by EBSD analysis for the untreated specimen and the heat-treated one. The result of the EBSD analysis showed that a noisy IPF map was obtained in the untreated specimen. This means that the untreated specimen has a certain strain. In contrast, in the heat-treated specimen, the clear IPF map that consists of grains grown by recrystallization was obtained. These results correspond to the changes in the grain size and the residual stress by heat treatment shown in Table 3 and Table 4. The deviation of the crystal orientation distribution such as a texture was not observed in the heat-treated specimen shown in Figure 3b.

### 3.2. Tensile Properties

**Figure 4:**
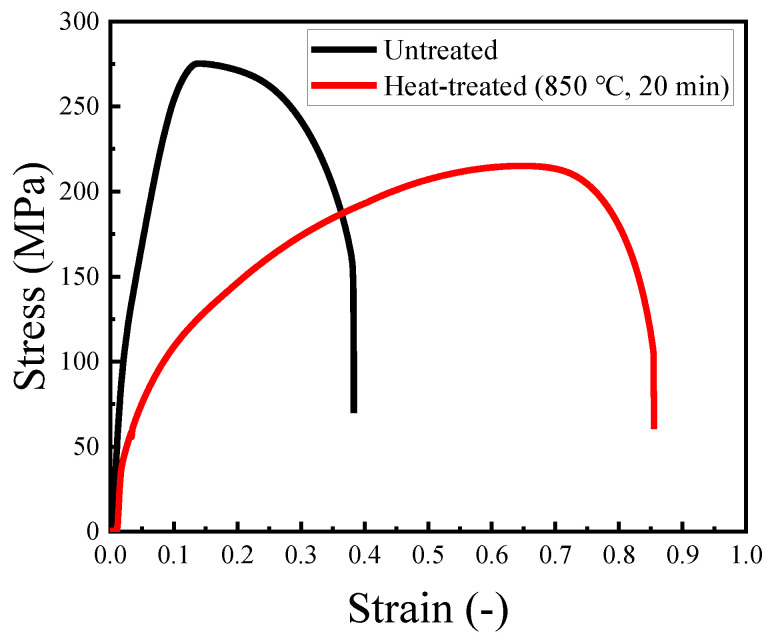
Typical stress–strain curves of untreated and heat-treated specimens.

Figure 4 shows typical stress–strain curves of the untreated specimen and the heat-treated one. It was found that the tensile strength was decreased and the elongation was increased by heat treatment accompanying recrystallization. In this study, three specimens were tested in each specimen types. Table 5 shows averages of tensile strength, 0.2% proof stress and elongation. From comparison of the average values, it was also confirmed that the tensile strength and the 0.2% proof stress were decreased, and the elongation was increased by heat treatment. Considering the results of the tensile test shown in Figure 4 and Table 5, the stress amplitude of the low cycle fatigue test was set to 50–200 MPa in this study.

### 3.3. Low Cycle Fatigue Properties

#### 3.3.1. Result of Low Cycle Fatigue Test

**Figure 5:**
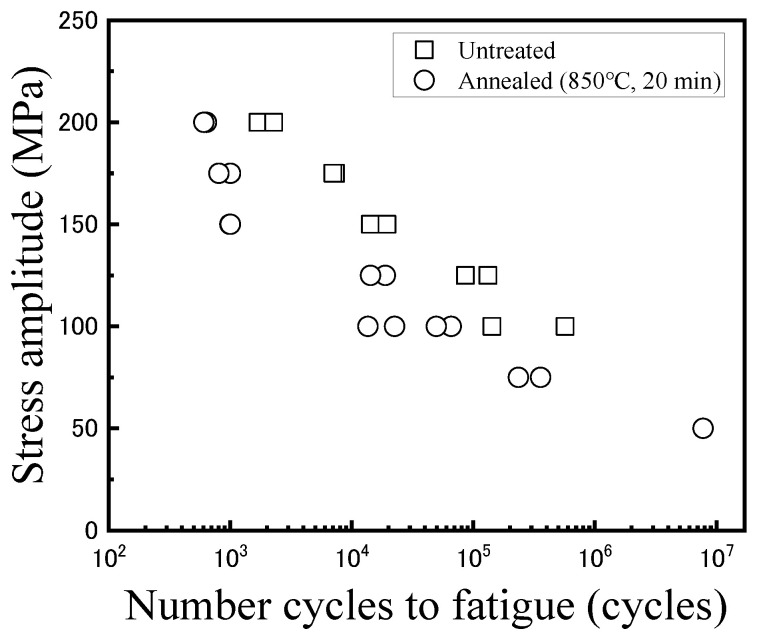
Low cycle fatigue test results.

**Figure 6:**
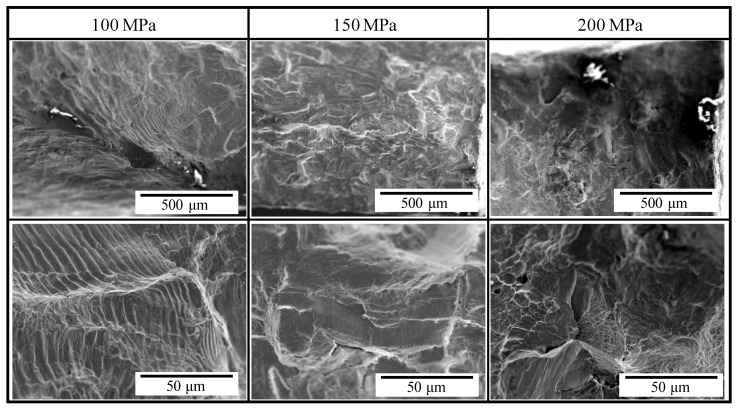
Secondary electron images of fracture surfaces of untreated specimens after the low cycle fatigue test. Bottom images are magnified images of upper ones.

**Figure 7:**
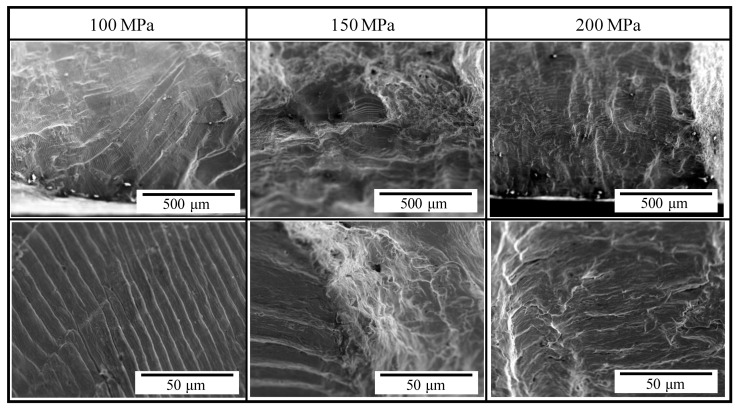
Secondary electron images of fracture surfaces of heat-treated specimens after the low cycle fatigue test. Bottom images are magnified images of upper ones.

Figure 5 shows the results of the low cycle fatigue test. The fatigue life was found to be decreased by heat treatment accompanying recrystallization. The observation results of fracture surfaces are shown in Figure 6 and Figure 7. In both figures, the areas that were estimated to be crack initiation areas by fracture surface observation are shown, and the bottom images are magnified images of upper ones. In the untreated specimens, striation that is a characteristic pattern in the fatigue failure was observed when the stress amplitude was 100 MPa. Under the stress amplitude condition, the fatigue life became more than 10^5^ cycles. Thus, striation was confirmed to be observed in the high cycle fatigue region exceeding 10^5^ cycles. In contrast, a river pattern and quasicleavage fracture were mainly observed in the fracture surface when the stress amplitude was more than 150 MPa, and the fatigue life became less than approximately 10^4^ cycles. As shown in Figure 4, when the stress more than 150 MPa is loaded, strain arrives at a few percentages or more. In such conditions, corresponding to the low cycle fatigue region, a few percent plastic deformation occurs accompanying the river pattern and the quasicleavage fracture, and thus, the fracture mode without striation was observed.

In the heat-treated specimens, striations were observed in the fracture surfaces regardless of the stress amplitude. When the stress amplitude was less than 100 MPa, and the fatigue life became more than 10^4^ cycles, striations were observed clearly in almost the whole of the crack initiation area. In addition, river patterns and quasicleavage fracture were observed with striations in the fracture surfaces when the stress amplitude was more than 150 MPa. Under such stress amplitude conditions, the fatigue life became less than 10^3^ cycles. As well as the untreated specimens, the tendency for the brittle fracture including the river pattern and quasicleavage to increase with an increase in the stress amplitude was found. Moreover, the width of striation was confirmed to be larger with the increase in the stress amplitude. This is because the strain is drastically increased with an increase in the loaded stress, as shown in Figure 4. Since the heat-treated specimen was softened by recrystallization, the brittle fracture including the river pattern and quasicleavage was considerably restrained even in strain at the level of dozens of percent, and thus, fracture surfaces with striations, river patterns, and quasicleavage were observed.

Compared with the width of striations in the case of stress amplitude of 100 MPa, the width of striation in the heat-treated specimen was larger than that in the untreated one. This means that the plastic strain amplitude imposed to the heat-treated specimen during the fatigue test was larger than that of the untreated one under the same stress amplitude condition. This is because the heat-treated specimen was softened by recrystallization, as shown in Figure 4. Since the plastic strain in the heat-treated specimen was increased compared with that in the untreated specimen under the same stress amplitude condition, the low cycle fatigue life of the C1020 oxygen-free copper was degraded by heat treatment, as shown in Figure 5.

Table 6 shows the measurement result of residual stress in the center of specimens after the low cycle fatigue test. Compared with Table 4 and Table 6, in the untreated specimen, the residual stress was changed from compressive stress to tensile stress when the stress amplitude was 100 MPa. The fractured strain was 15% tensile strain, and thus, the residual stress remained in the same direction. In contrast, in the heat-treated specimens, both tensile and compressive stress were detected depending on the specimen when the stress amplitude was 100 MPa. The specimen with 30% tensile fractured strain showed the tensile residual stress, and the specimen with 5% compressive fractured strain showed the compressive residual stress. It was also confirmed that the compressive residual stress after the low cycle fatigue test was increased compared with that before the test, as shown in Table 4. In the case of the stress amplitude of 50 MPa, although the fractured strain was approximately zero, the compressive residual stress was remained, and it was higher than that before the test. The result indicates that the strain was accumulated in the specimen by the fatigue cycle. In this way, the residual stress in the center of the specimen was mainly dependent on the deformation state of the specimen.

#### 3.3.2. Estimation of Fatigue Damage Behavior by EBSD Analysis

**Figure 8:**
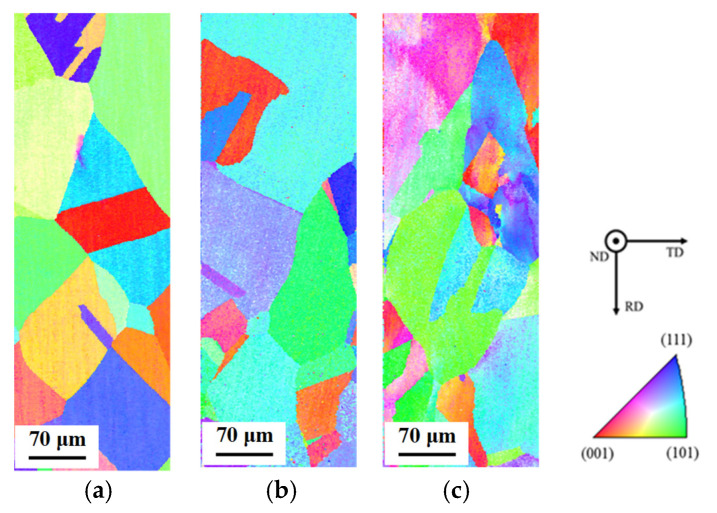
IPF maps of heat-treated C1020 specimens after low cycle fatigue test. (**a**) Stress amplitude: 50 MPa (fatigue cycle: 7,786,201 cycles); (**b**) stress amplitude: 75 MPa (fatigue cycle: 235,144 cycles); (**c**) stress amplitude: 100 MPa (fatigue cycle: 49,676 cycles).

In this study, fatigue damage behavior was investigated by EBSD analysis. Figure 8 shows IPF maps of the center of the heat-treated specimens after the low cycle fatigue test. In the IPF maps, the grain boundary with misorientation more than 15° was defined as the high-angle grain boundary. When the stress amplitude was 100 MPa, the gradation appeared in several grains. Those grains were found to be deformed due to strain accumulated by the fatigue cycle.

**Figure 9:**
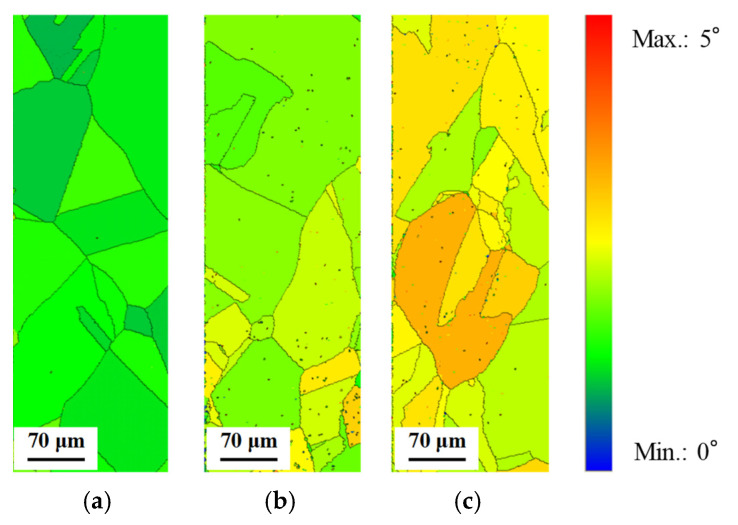
Grain average misorientation (GAM) maps of heat-treated C1020 specimens after low cycle fatigue test. (**a**) Stress amplitude: 50 MPa (fatigue cycle: 7,786,201 cycles); (**b**) stress amplitude: 75 MPa (fatigue cycle: 235,144 cycles); (**c**) stress amplitude: 100 MPa (fatigue cycle: 49,676 cycles).

**Figure 10:**
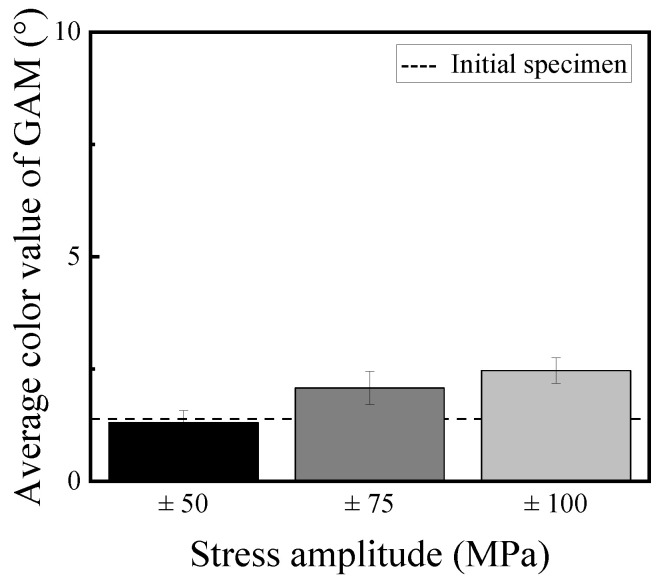
Relationship between GAM value and stress amplitude.

Figure 9 shows the grain average misorientation (GAM) maps for the IPF maps. The GAM map is the average misorientation in the grain, which is obtained by dividing the sum of the misorientation of all of the boundaries between pixels by the number of boundaries [18]. Figure 9 reveals that the GAM value was increased with an increase in the stress amplitude. Figure 10 shows the change of the GAM value with changing the stress amplitude compared with that of the initial specimen. The GAM value was confirmed to be scarcely changed compared with the initial specimen when the stress amplitude is 50 MPa. The GAM value was also confirmed to be increased with the increase in the stress amplitude from Figure 10. Although the difference in the misorientation that depended on each grain could be detected by the GAM maps, the misorientation in the grain which corresponds to the fatigue damage in the grain could not be detected.

**Figure 11:**
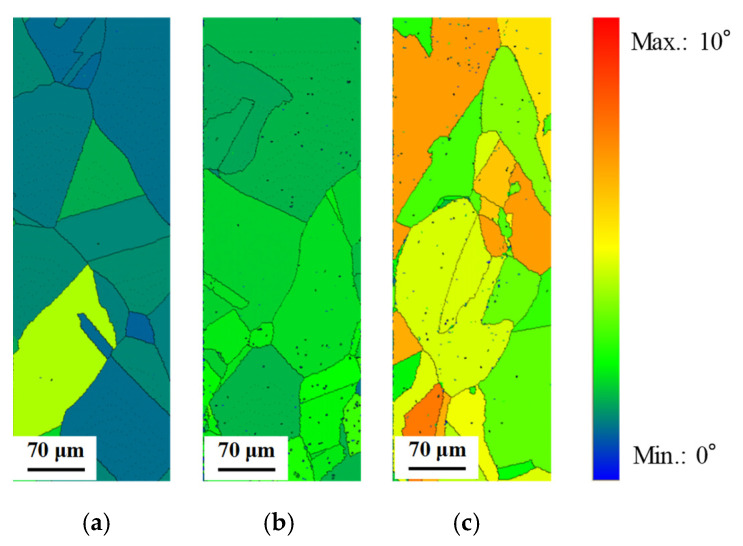
Grain orientation spread (GOS) maps of heat-treated C1020 specimens after low cycle fatigue test. (**a**) Stress amplitude: 50 MPa (fatigue cycle: 7,786,201 cycles); (**b**) stress amplitude: 75 MPa (fatigue cycle: 235,144 cycles); (**c**) stress amplitude: 100 MPa (fatigue cycle: 49,676 cycles).

**Figure 12:**
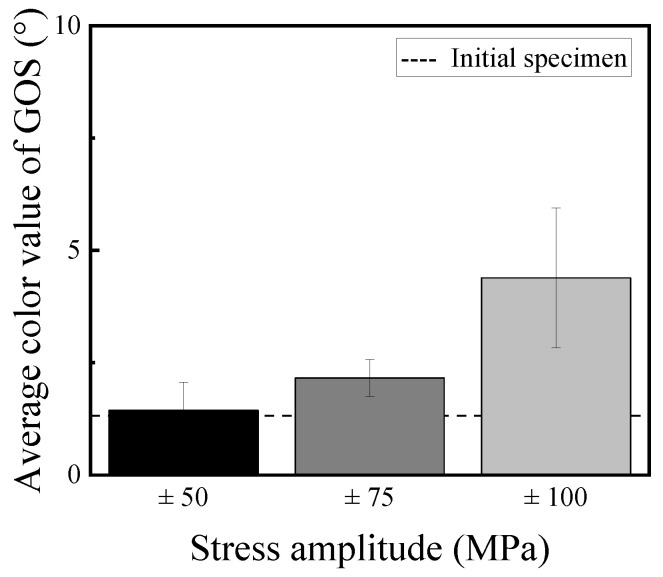
Relationship between GOS value and stress amplitude.

Figure 11 shows grain orientation spread (GOS) maps for the IPF maps. To obtain the GOS map, one pixel in the grain is set as the reference point. Next, the misorientation between another pixel in the grain and the reference point is investigated for all pixels in the grain. The average of the misorientations is defined as GOS by a grain unit [18]. From Figure 11, the GOS value is also increased with an increase in the stress amplitude. However, the distribution of the value in the observation area was slightly different between both GAM and GOS maps in the stress amplitude of 100 MPa. This seems to be dependent on the definition of both maps. The misorientation in the grain could not be also detected by the GOS map. Figure 12 shows the change of the GOS value with changing the stress amplitude compared with that of the initial specimen. As well as the GAM map, the GOS value was scarcely changed compared with the initial specimen when the stress amplitude was 50 MPa. In addition, the GOS value was increased with the increase in the stress amplitude. In the case of the stress amplitude of 100 MPa, the unevenness of the GOS values in the observation area became large. This result indicates that it is feasible to evaluate the fatigue damage of the specimen by the evenness of the GOS value.

**Figure 13:**
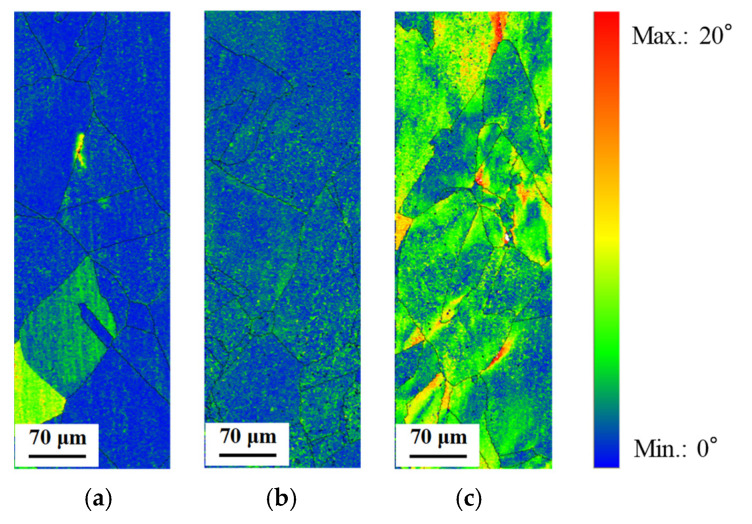
Grain reference orientation deviation (GROD) maps of heat-treated C1020 specimens after low cycle fatigue test. (**a**) Stress amplitude: 50 MPa (fatigue cycle: 7,786,201 cycles); (**b**) stress amplitude: 75 MPa (fatigue cycle: 235,144 cycles); (**c**) stress amplitude: 100 MPa (fatigue cycle: 49,676 cycles).

Figure 13 shows grain reference orientation deviation (GROD) maps for the IPF maps. The GROD map is created using the kernel average misorientation (KAM) map. Generally, hexagonal pixels are used in the EBSD analysis. To obtain the KAM map, the six misorientations between a certain one pixel and adjacent pixels are investigated, and the average of them is obtained by a pixel unit [19]. To obtain the GROD map, the misorientation between another pixel and the pixel with the minimum KAM value is investigated in the grain. The GROD map is obtained by a pixel unit as well as the KAM map [19]. Compared with Figure 8 and Figure 13, it was found that the gradation in the grain shown in the IPF map corresponds well with the gradation in the GROD map when the stress amplitude is 100 MPa. This result indicates that the GROD map is effective to investigate the fatigue damage degree in the grain.

**Figure 14:**
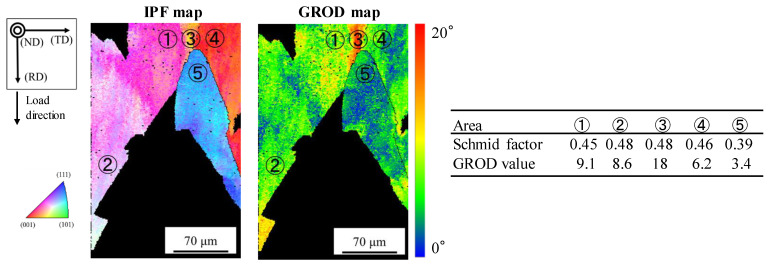
IPF map and GROD map of the area that shows the high GROD value in Figure 13c, and Schmid factor and GROD values for each area shown in the figure.

Figure 14 shows the IPF map and the GROD map of the area that shows the high GROD value in Figure 13c. The Schmid factor and GROD values for each area shown in Figure 14 are also indicated in the figure. The areas of ①, ②, and ③ exist in the same grain, and the areas of ④ and ⑤ exist in other grains, respectively. From Figure 14, the grain including ①, ②, and ③ and the grain including ④ was found to have high Schmid factors near 0.5. On the other hand, the grain including ⑤ had a smaller Schmid factor. Thus, sliding deformation occurs easily in the grains including ③ and ④ compared with the grain including ⑤. Therefore, stress concentration occurred in the vicinity of the grain boundary between the grain including ③ and the grain including ④, and thus, the large GROD value generated in the area of ③ in the vicinity of the grain boundary. The similar large GROD value was also shown in the area of ③ in the vicinity of the triple point of the three grains. In this way, high GROD values were detected in the vicinity of the grain boundaries and the triple points of grains in which stress concentration occurred easily.

From the above-mentioned results, the GROD value was clarified to be effective to investigate the fatigue damage degree in the grain of the oxygen-free copper by low cycle fatigue.

**Figure 15:**
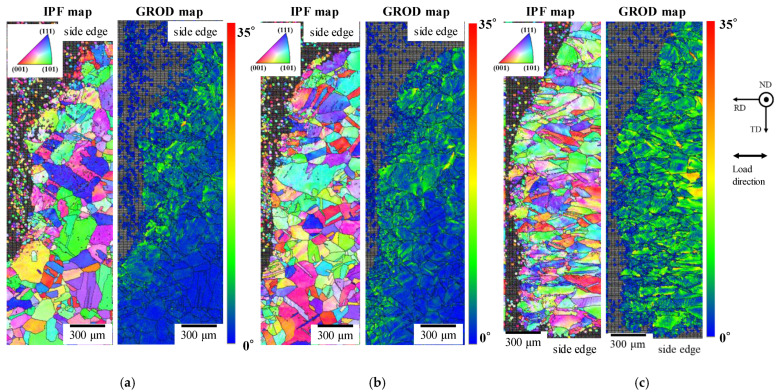
IPF maps and GROD maps of cross-sections of fractured areas of heat-treated specimens after low cycle fatigue test. (**a**) Stress amplitude: 50 MPa (fatigue cycle: 7,786,201 cycles); (**b**) stress amplitude: 75 MPa (fatigue cycle: 235,144 cycles); (**c**) stress amplitude: 100 MPa (fatigue cycle: 49,676 cycles).

Figure 15 shows IPF maps and GROD maps of cross-sections of fractured areas after a low cycle fatigue test. The figure shows the regions that were assumed as crack initiation sides by the observation of the fractured surfaces. Compared with the GROD maps of the centers of the heat-treated specimens shown in Figure 13, the GROD values were found to be increased in the fractured areas. In addition, the region with high GROD values was expanded with an increase in the stress amplitude. The maximum GROD values were 28.4°, 27.8°, and 31.5° for the specimens investigated at stress amplitudes of 50, 75, and 100 MPa, respectively. The results indicate that the crack generated in the area in which the grains were deformed and the GROD value reached approximately 28°. Such a GROD value is possibly used to investigate the fatigue damage degree in the grain, the crack initiation behavior, and the crack progress behavior. However, the relationship between the GROD value and the plastic strain accumulated in the grain has not been clarified yet. Thus, further study is required to make the GROD value an index for the fatigue damage degree in the grain of the oxygen-free copper by low cycle fatigue.

## 4. Conclusions

In this study, the effects of heat treatment by a vacuum brazing process on tensile properties and low cycle fatigue properties of the oxygen-free copper were investigated. Moreover, the fatigue damage behavior was investigated by EBSD analysis. The results obtained are summarized as follows.

(1) The heat treatment caused the grain growth and relaxation of strain by recrystallization, and thus, the residual stress in the copper was reduced. Due to the grain growth by the heat treatment, the tensile strength and the 0.2% proof stress were decreased, and the elongation was increased.

(2) The plastic strain was increased in the heat-treated specimen compared with that in the untreated specimen under the same stress amplitude condition, and thus, the low cycle fatigue life of the oxygen-free copper was degraded by heat treatment. Striation was observed in the crack initiation region of the fractured surface in the case of the stress amplitude less than 100 MPa regardless of the presence of the heat treatment. With an increase in the stress amplitude, the river pattern and the quasicleavage fracture were mainly observed in the fracture surface of the untreated specimens and were observed with striations in the fracture surfaces of the heat-treated specimens.

(3) The residual stress in the center of the specimen after the low cycle fatigue test was changed mainly depending on the deformation state of the specimen.

(4) From the results of the EBSD analysis for the centers of the specimens fractured by the low cycle fatigue test, the GROD value was found to be effective to investigate the fatigue damage degree in the grain of the oxygen-free copper by low cycle fatigue.

(5) From the results of the EBSD analysis for the fractured areas of the heat-treated specimens after the low cycle fatigue test, the crack was found to be generated in the area in which the grains were deformed and the GROD value reached approximately 28°.

## Figures and Tables

**Table 1:** Chemical composition of C1020 used in this study.

Elements	Cu	Pb	Fe	Sn	Zn	Al	Mn	Ni	P
mass%	≧99.96	-	-	-	-	-	-	-	-

**Table 2:** Composition of etching solution used in this study.

Ammonium Chloride (g)	Sulfuric Acid (mL)	Nitric Acid (mL)	Chromium Trioxide (g)	De-Ionized Water (mL)
1.88	12.5	12.5	10	475

**Table 3:** Average grain size estimated from Figure 2.

Heat Treatment Conditions	Average Grain Size (μm)
Untreated	129.9
850 °C, 20 min	182.2

**Table 4:** Residual stress estimated using a micro-area X-ray residual stress measurement system.

Heat Treatment Conditions	Residual Stress (MPa)
Untreated	−86.9
850 °C, 20 min	−70.3

**Table 5:** Results of tensile test.

Heat Treatment Conditions	Tensile Strength (MPa)	0.2% proof Stress (MPa)	Elongation (%)
Untreated	281	73.6	41.5
Heat-treated (850 °C, 20 min)	212	42.0	80.8

**Table 6:** Residual stress estimated after low cycle fatigue test using a micro-area X-ray residual stress measurement system.

Heat Treatment Conditions	Stress Amplitude (MPa)	Fatigue Cycle (cycles)	Fractured Strain (%) +: Tensile, −: Compressive	Residual Stress (MPa)
Untreated	100	142,214	+15	+50.4
850 °C, 20 min	100	49,676	+30	+59.6
850 °C, 20 min	100	22,503	−5	−117.7
850 °C, 20 min	50	7,786,201	≈0	−142.2

## Data Availability

Data are contained within the article.
